# Functional blockade of α_5_β_1 _integrin induces scattering and genomic landscape remodeling of hepatic progenitor cells

**DOI:** 10.1186/1471-2121-11-81

**Published:** 2010-10-19

**Authors:** Luciano Vellón, Félix Royo, Rune Matthiesen, José Torres-Fuenzalida, Alicia Lorenti, Luis A Parada

**Affiliations:** 1 Cytogenomics, CIC bioGUNE-CIBEREHD, Par. Tec. Bizkaia Ed. 801 A, 48160 - Derio, Spain; 2 IPATIMUP - Institute of Molecular Pathology and Immunology, University of Porto, Portugal; 3 Institute for Basic Sciences and Experimental Medicine, Hospital Italiano, Potosi 4240, 1199 - Buenos Aires, Argentina; 4 Unidad de Traducción Clínica - Ingeniería de Tejidos, Austral University, Pte Perón 1500, 1635-Pilar, Argentina; 5 Institute of Experimental Pathology, Faculty of Health Sciences, National University of Salta, Bolivia 5010, 4400-Salta, Argentina

## Abstract

**Background:**

Cell scattering is a physiological process executed by stem and progenitor cells during embryonic liver development and postnatal organ regeneration. Here, we investigated the genomic events occurring during this process induced by functional blockade of α_5_β_1 _integrin in liver progenitor cells.

**Results:**

Cells treated with a specific antibody against α_5_β_1 _integrin exhibited cell spreading and scattering, over-expression of liver stem/progenitor cell markers and activation of the ERK1/2 and p38 MAPKs signaling cascades, in a similar manner to the process triggered by HGF/SF1 stimulation. Gene expression profiling revealed marked transcriptional changes of genes involved in cell adhesion and migration, as well as genes encoding chromatin remodeling factors. These responses were accompanied by conspicuous spatial reorganization of centromeres, while integrin genes conserved their spatial positioning in the interphase nucleus.

**Conclusion:**

Collectively, our results demonstrate that α_5_β_1 _integrin functional blockade induces cell migration of hepatic progenitor cells, and that this involves a dramatic remodeling of the nuclear landscape.

## Background

Cell scattering is a physiological process executed by stem and progenitor cells during embryonic liver development and postnatal organ regeneration. Metastasis seems to arise from the same genetic program that instructs cells to detach, adhere, and migrate through extracellular matrices, crossing tissue boundaries and escaping death due to an unsuitable tissue context [1]. The Hepatocyte Growth Factor/Scattering Factor 1 (HGF/SF1) is the paradigmatic example of a molecule that induces cell scattering with optimal spatial and chronological coordination. This process takes place through a complex network of signaling pathways triggered by the HGF/SF1 tyrosine kinase receptor, Met, which includes the Grb2-Ras-Mitogen Activated Protein Kinases (MAPK), the PI-3'K, and the Signal Transducer and Activator of Transcription (STAT) cascades [2]. Integrins are thought to be essential for cell migration and penetration of the basement membrane, in addition to playing a major role in cellular adhesion to the extracellular matrix (ECM) and certain cell surface proteins. These adhesion receptors also convey a series of mechanical and biochemical extracellular stimuli in signaling cascades that favor cell migration and proliferation [3,4]. Interestingly, growth factor and integrin-emanating signals can interact to promote cell migration. For instance, c-Met signaling can be modulated by the α_6_β_4 _integrin when co-expressed on the cell surface [5], and HGF/SF1, conversely, can regulate the adhesive status and aggregation rate of α_v_β_3 _integrin in epithelial cells [6].

The genome is highly organized within the cell nucleus [7]. Indeed, chromosomes and genes exhibit cell type specific preferential positioning, and this non-random distribution of genetic elements in the interphase nucleus is related to genome function [8]. Genome organization has been broadly investigated, in particular during cell differentiation and tumorigenesis. For example, the stem cell specific genes *Nanog *and *Oct4 *acquire differential positioning in the nucleus as their expression levels change during differentiation of human embryonic stem cells [9]. Additionally, changes in the spatial distribution of cancer related genes have been shown in a cell-model of breast cancer during induced malignant evolution [10]. However, much still remains to be elucidated regarding the genomic events associated with cell migration.

MLP29 cells are murine liver progenitor cells that respond to HGF/SF1 treatment with a well characterized sequence of events that resemble the cell invasion program, including cell scattering, migration, proliferation, and tubular morphogenesis [11]. Considering that α_5_β_1 _integrin is one of the main ECM receptors of hepatocytes, and that changes in integrin-mediated contacts between the cell and ECM are necessary for cell migration [12], we speculate that functional blockade of α_5_β_1 _integrin may result in cell migration, and that the changes in the cell microenvironment will be sensed and transduced into specific nuclear responses. Here, we investigated the molecular and cellular genomic events associated with cell migration triggered by the functional blockade of α_5_β_1 _integrin in liver progenitor cells.

## Results

### Integrin expression profile and adhesion properties of hepatic progenitor and HCC cells

**Figure 1:**
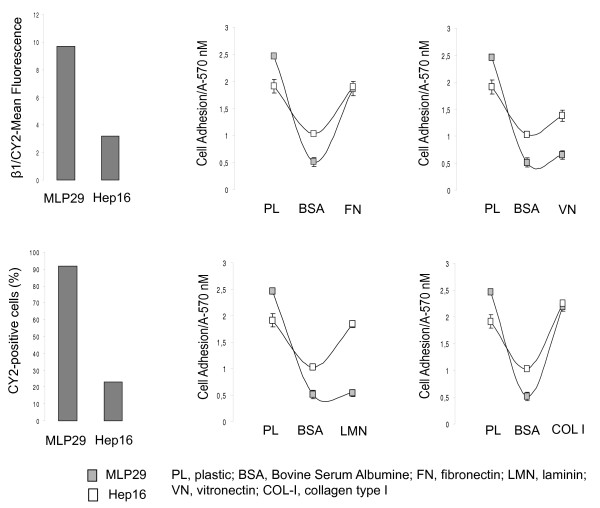
**Β1-integrin expression and adhesive properties of MLP29 and Hep16 cells**. (A) Quantitative analysis by flow cytometry revealed that most (90%) of cells were positive for β_1 _staining and that the intensity levels (Geo Mean fluorescence) of β_1 _expression was higher in MLP29 than in Hep16 cells. The experiment was performed three times with similar results. (B) MTT assay was used to assess MLP29 and Hep16 cell adhesion capacity to different components of the extracellular matrix, including FN, VN, LMN and COL I. To prevent nonspecific cell adhesion, plates were blocked with BSA. Cell adhesion to the different substrates was estimated by measuring the optic density at A570 nm. The data presented summarize the mean (±SD) of three independent experiments, each performed in triplicate.

We first assessed the cell membrane expression of β_1 _integrins in MLP29 and Hep16 cells by flow cytometry and immunofluorescence analyses. Cytometric analyses showed that this integrin sub-unit is expressed in MLP29 and Hep16 cells. However, flow cytometric detection of β_1 _levels demonstrated significantly higher levels of β_1 _integrin-types in MLP29 cells compared to Hep16 cells, used as a control because of their low β_1 _integrin expression levels (Figure 1A). To determine the adhesive capacity to different components of the ECM, we performed cell adhesion tests to FN, VN, LMN and COL-I using the MTT assay. HCC cells exhibited high adhesive capacity to all these components of the ECM, whereas, MLP29 cells displayed high adhesion to FN and COL-I, and low adhesion to VN and LMN (Figure 1B), thus making the MLP29 cellular model suitable for the study of β_1 _integrin-mediated events.

### α_5_β_1 _integrin functional blockade induces cell scattering and migration in hepatic progenitor cells

**Figure 2:**
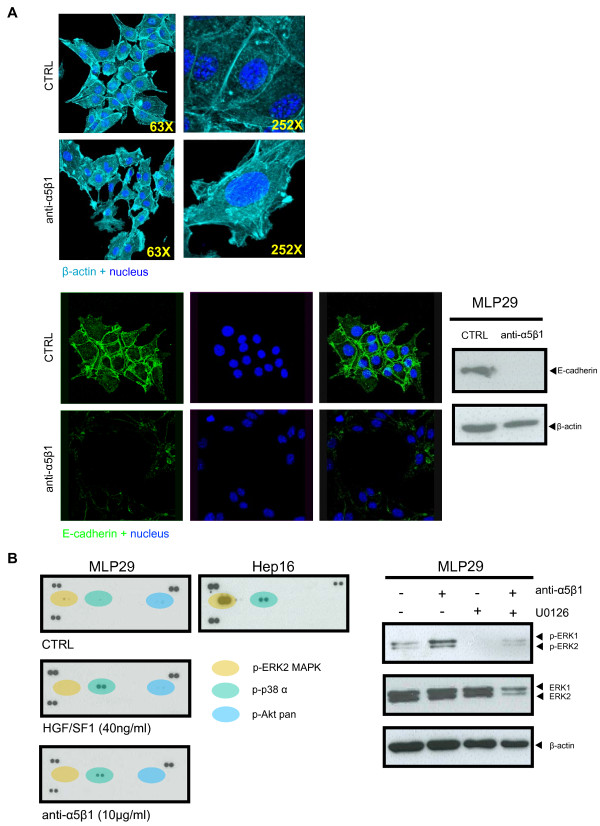
**α_5_β_1 _integrin functional block induced spreading and scattering of MLP29 hepatic progenitor cells**. (A) Immunofluorescent detection of F-actin in MLP29 cells showed that the cells grow in packed islands, while after the treatment with a specific α_5_β_1_-functional blocking antibody the cells undergo spreading and scattering. Higher magnification (252×) of untreated and treated MLP29 cells shows the characteristic actin microfilaments reorientation associated with cytoskeleton re-organization. Immunofluorescent and immunoblot detection of E-cadherin in MLP29 cells following α_5_β_1 _functional blockade showed that the expression levels of E-cadherin decreased at the cell-cell junctions in cells treated with the specific functional blocking antibody against α_5_β_1_, which was corroborated by immunoblot analysis. (B) Representative images of the phospho-MAPKs arrays. Activation of the different members of the MAPKs family was identified by means of a key provided with the kit. MLP29 cells were treated with the α_5_β_1 _functional blocking antibody or HGF/SF1, in the presence or absence of the specific MEK inhibitor U0126. Cells were lysed and total protein (20 μg) was resolved by SDS-PAGE and analyzed by immunoblot for ERK1/ERK2 MAPK and phospho-ERK1/ERK2 MAPKs. Blots were then reprobed with an antibody for β-actin as a control for protein loading. Results are representative of at least three independent experiments.

Considering that integrin α_5_β_1 _is one of the main ECM receptors of hepatocytes, we wondered whether specific disruption of the α_5_β_1_-mediated cell-ECM interactions would trigger invasive-like cell growth. In order to substantiate our hypothesis, MLP29 cell cultures were treated with a specific antibody against α_5_β_1 _integrin and HGF/SF1 as control, owing to its capacity to induce cell scattering and migration. By microscopic inspection, we observed that untreated MLP29 cells grow in tightly packed patches, mimicking the structure of an epithelial sheet, whereas blocking α_5_β_1 _caused the loss of cell-cell junctions and induced cell spreading throughout the culture dish, similar to the cell scattering induced by HGF/SF1. These morphological changes involved reorganization of the cell cytoskeleton, from cortical F-actin structures to parallel bundles disposed along the axis of the cellular prolongations (Figure 2A). Moreover, immunoblot and immunofuorescent detection showed that functional blockade of α_5_β_1 _integrin induced down-regulation of E-cadherin, demonstrating that effectively the process involved cell-cell dissociation (Figure 2A).

**Figure 3:**
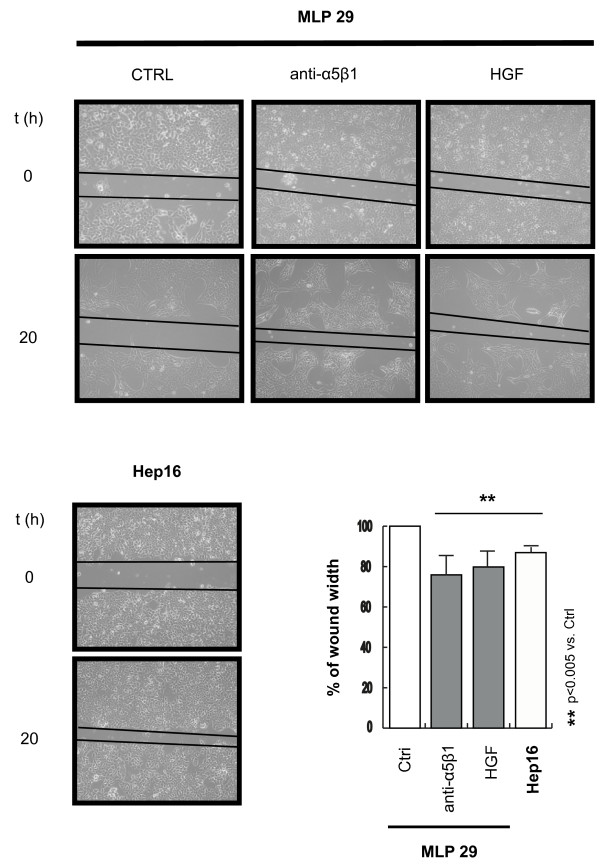
**α5β1 integrin functional blockade induced migration of MLP29 hepatic progenitors**. α_5_β_1 _functional blockade induced morphological modifications and triggered cell motility in MLP29 cells, in a manner comparable to HGF/SF1. Cell migration was calculated as the mean of six different measurements along the scratch and is expressed as percentage of the wound width covered by the migrating cells. The data presented summarize the mean (±SD) of three independent experiments.

To specifically test cell migration we performed the wound healing and the transwell assay. In the wound healing assay, the same fields on a confluent cell monolayer were pictured right after scratching (0 hours) and 20 hours later. Computational analysis of the images showed that MLP29 cells treated with the α_5_β_1 _function-blocking antibody migrated towards the scratch faster than the control cells (p < 0.005). HGF/SF1 also induced migration, but to a lesser extent (Figure 3). In the transwell assay, MLP29 cells treated with HGF/SF1 or anti-α_5_β_1 _integrin antibody were placed on top of filters coated with ECM formulations, but migration through filters was not statistically significant after 72 hours. These results suggest that different mechanisms for cell migration may be used in these two types of assays (see below). We further characterized this process by determining the expression levels of β_1 _and β_3 _integrin sub-units, which are known to be associated with the invasive capacity of several epithelial tumor types. Moreover, we assessed the level of EpCAM, AFP and CK19, all biomarkers expressed in the liver stem cell niche during liver regeneration [13]. Immunofluorescence with antibodies against these proteins showed that the expression level is higher after both treatments (Additional file 1). In addition, these changes were associated with an increase in metabolic activity (Additional file 2). Furthermore, flow cytometric analysis of DNA content showed that α_5_β_1 _functional blockade did not induce drastic changes in the progression of MLP29 cells through the cell cycle (Additional Files 3 and 4).

### α_5_β_1 _integrin functional blockade of hepatic progenitor cells triggers cell signaling pathways involved in cell motility

We checked the activation status of the different members of the MAPKs family in response to α_5_β_1 _functional blockade or stimulation with HGF/SF1. Using a MAPK array kit, we determined that both treatments induced the long-term activation (twenty hours) of p38 MAPK, partly matching the hyperactivation of the ERK1/2 and p38 MAPKs cell transduction cascades of the highly invasive Hep16 HCC cells (Figure 2B). Activation of ERK 1/2 MAPKs was also observed, however, the peak of this kinase phosphorylation occurred two hours after α_5_β_1 _integrin functional blockade. These effects were reversed by the MEK pharmacological inhibitor U0126 (Figure 2B).

### Gene expression profile of migrating liver progenitor cells

To characterize the global genome transcriptional response to functional blockade of α5β1 integrin, we performed GeneChip array analyses (Affimetrix^®^, Mouse430A_2) of well annotated genes. The data from three independent experiments were collectively analyzed using the R package "AFFYLMGUI" (http://www.bioconductor.org). Each experiment included RNA samples from MLP29 cells subjected to functional blockade of α_5_β_1 _integrin, HGF stimulation, and untreated control cells. A total of 2671 genes changed their expression level during cell spreading induced by α5β1 functional blockade, whereas only 191 genes changed their transcriptional status after HGF/SF1 stimulation, when a p-value <0.05 was considered significant in the statistical comparisons between treated and control cells. Because genes are unequally distributed on chromosomes (gene density), we calculated the ratio between the number of differentially expressed genes per chromosome and the total number of genes mapping to the same chromosome (http://www.ensembl.org/index.html). This analysis showed that de-regulation of genes is evenly distributed throughout the genome. Gene ontology (GO) enrichment analysis demonstrated that during cell migration induced by α_5_β_1 _functional blockade, more genes were up-regulated than down-regulated. Most of the up-regulated genes were within the Biological Processes (BP) and Cellular Components (CC) categories (Table 1; Additional File 5). The number of up-regulated genes, found in many categories, can be narrowed down to seven, and up-regulation in the expression of the Transforming Growth Factor-β2 (*Tgfb2*) and Hypoxia-Induced Factor 1 alpha (*Hif1a*) genes appear to be involved in many of the observed cellular responses (Table 2 and Additional file 6). Down-regulations occurred more frequently within the Molecular Function (MF) compartment, specifically genes involved in the regulation of chromatin stability, such as *Sfpq*, *Dido1* and *Sap18* (Additional file 7).

**Table 1:** Most significant up-regulations in MLP29 cells upon α_5_β_1 _integrin functional blockade

Biological Processes (BP)				
**GO description**	**Main Category**	**# array**	**#DEGs**	**P(GO)**

Cell growth	P	10	3	1,15E-05
Aromatic compound metabolic process	P	40	4	2,71E-05
Response to external stimulus	P	206	7	2,80E-05
Regulation of biological process	P	2644	25	6,66E-05
Growth	P	53	4	8,00E-05
Response to stress	P	435	9	9,32E-05
Cell proliferation	P	147	5	0,000407675
Biosynthetic process	P	320	7	0,000415342
**Cellular Components (CC)**				
**GO description**	**Main category**	**# array**	**#DEGs**	**P(GO)**
Extracellular region	C	263	8	1,62E-05
Extracellular space	C	1083	13	0,00059

**Table 2:** Most significantly up-regulated genes (p < 0.0005) in MLP29 cells upon α_5_β_1 _integrin functional blockade

Genes	Main category	GO-description
Tgfb2+	Cell growth	BP
Tgfb2+	Aromatic compound metabolic process	BP
Tgfb2+	Response to external stimulus	BP
Sfpq- Dido1- Ncoa5- Sap18- Traf3- Hif1a+ Maff+ Bnc1+ Tgfb2+ Gpx1+	Regulation of biological processes	BP
Tgfb2+	Growth	BP
Sfpq- Hif1a+ Tgfb2+ Gpx1+	Response to stress	BP
Tgfb2+	Cell proliferation	BP
Mgat2- Ncoa5- Nmt1- Hif1a+ Tgfb2+	Biosynthetic process	BP
Tgfb2+	Extracellular region	CC
Ide- Lman2- Vnn3+ Tgfb2+ Slpi+	Extracellular space	CC

**Figure 4:**
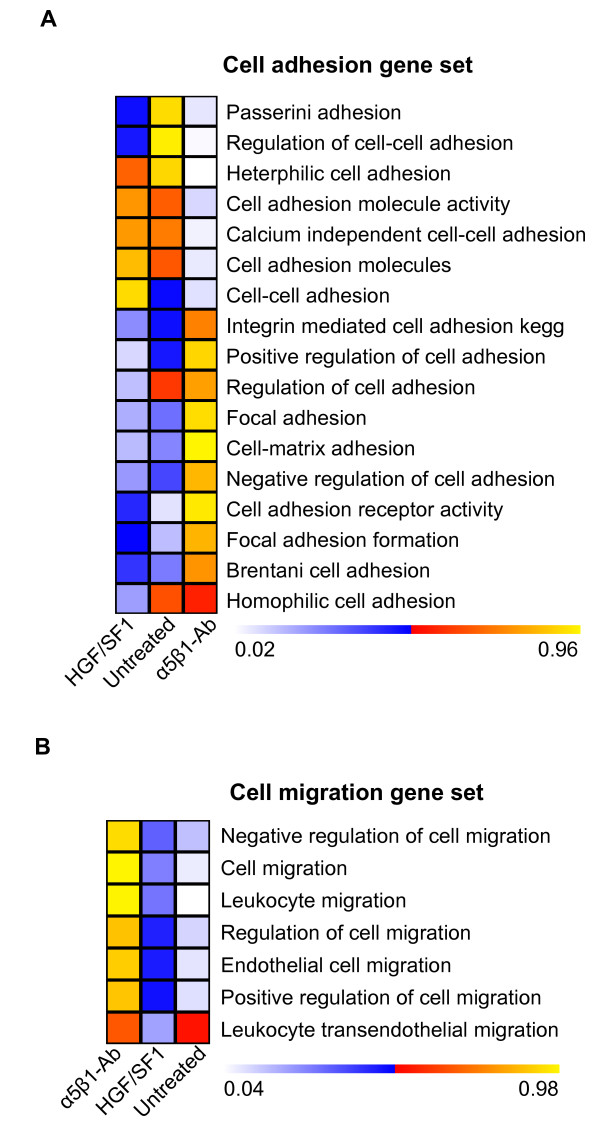
**Gene expression profiling of MLP29 hepatic progenitor cells after α_5_β_1 _integrin blockade and HGF/SF stimulation**. (A) (B) Plots showing the changes in the expression of gene sets involved in cell adhesion and migration, respectively. Data from three independent experiments were pooled and subjected to permutation analyses to assess the expression level changes of gene sets obtained from the Molecular Signatures Database. The resulting data are presented as color-encoded plots in which a p-value close to 1 indicates significant up-regulation, and a p-value close to 0 indicates a significant down-regulation.

Next we assessed the transcriptional response of gene sets involved in cell adhesion and migration. The results are presented in Figure 4 as color-encoded plots in which a p-value close to 1 indicates statistically significant higher mRNA levels of all genes included in the set, and a p-value close to 0 indicates significantly lower levels. The functional blockade of α_5_β_1 _induced up-regulation of several sets of genes involved in cell adhesion, whereas the response to HGF/SF1 stimulation was less pronounced, similar to untreated cells (Figure 4A). The gene sets involved in cell migration exhibited a pattern of gradual change in expression levels among the three types of samples. As expected, untreated cells exhibited significantly lower expression levels of these genes compared to the other two groups of treated cells, whereas the treatment with HGF/SF1 induced a slight increase, and α_5_β_1 _functional blockade resulted in a more pronounced up-regulation of cell-migration genes (Figure 4B), among them β_1 _and β_3 _integrins. However, before regarding the results obtained for HGF/SF1 as not significant (p-value 0.05 < p < 0.95), it must be recognized that the permutation analyses were done across all samples, including the α5β1 inhibition which displays much stronger regulation of cell migration genes. Most important, these results clearly demonstrate that α5β1 functional blockade triggers invasive-like cell migration.

### Distinct expression profile of chromatin-remodeling and transcription factors

**Figure 5:**
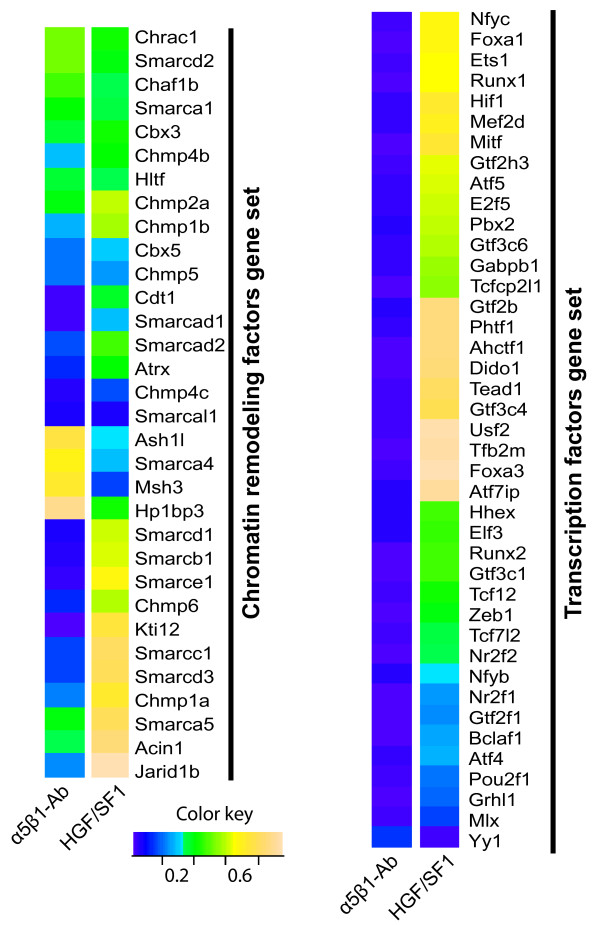
**Changes in the expression level of genes encoding chromatin remodeling and transcription factors in MLP29 cells during migration**. (A) Changes in the expression level of genes encoding chromatin remodeling factors were plotted according to their level of significance as a color-encoded map, in which a p-value close to 1 indicates significant up-regulation, and a p-value close to 0 indicates a significant down-regulation. (B) Changes in the expression level of transcription factors following the treatment with α_5_β_1 _integrin blocking antibody or with HGF/SF1 for 24 hours.

A total of 32 genes, belonging to the Smarc (SWI/SNF-related, matrix-associated, actin-dependent regulators of chromatin) family of chromatin remodeling factors were represented in the array and the analysis of their expression data revealed that functional blockade of α_5_β_1 _resulted in a larger number of differentially regulated genes than HGF/SF1 stimulation (Figure 5). We also analyzed the expression level of transcription factors, and our results demonstrate drastic changes in the expression level in integrin inhibited cells, whereas stimulation with HGF/SF1 presented a more restricted response (Figure 5). More importantly, the transcriptional responses to these two treatments are clearly distinct, indicating that the genomic effects exerted by the disruption of cell-ECM interactions differ from those induced by soluble regulatory factors (HGF/SF1), possibly because cells also apply traction to their integrin receptors, in addition to the integrin ligation-induced signaling.

### Cell scattering is associated with nuclear architecture remodeling

**Figure 6:**
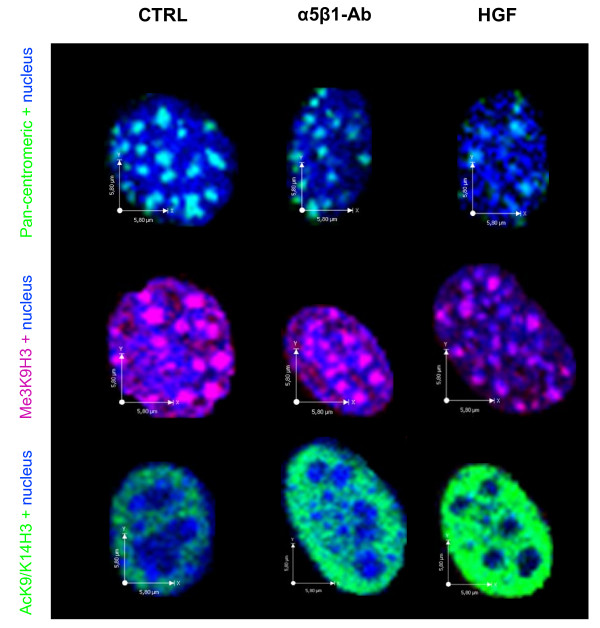
**Change of the nuclear organization of MLP29 hepatic progenitor cells**. Quantitative FISH was performed with a pan-centromeric probe. Computational analysis of the spatial organization of chromocenters showed marked variations in their average volume, whereas the nuclear volume remained unchanged in MLP29 cells following α_5_β_1 _blockade or stimulation with HGF/SF1. 3D computational reconstruction of the image stacks and quantitative analysis showed that the average fluorescence intensity of the Me3H3K9 foci remained unaltered. However, the number of foci increased concomitantly with a reduction of their volume after both types of treatments. Immunofluorescent detection of Ac3K9/14 H3, on the other hand, revealed that cell scattering is associated with an increase in the level of acetylation of histone H3 at K9.

**Figure 7:**
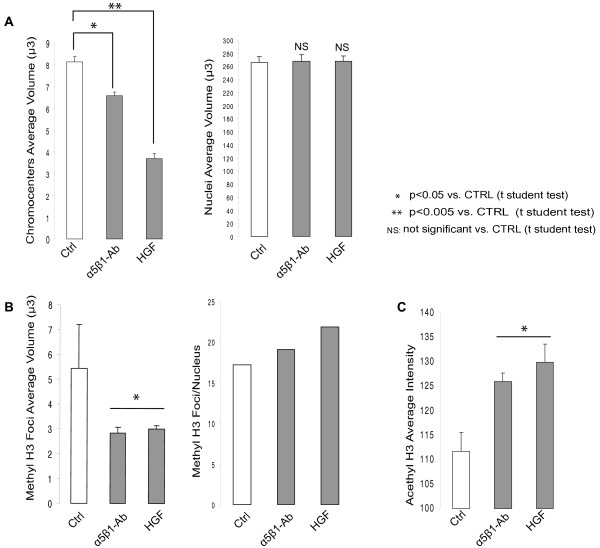
**Change of the nuclear organization of MLP29 hepatic progenitor cells**. (A) Bar graphs summarize the mean (±S.E.) volume of at least 1000 chromocenters analyzed and the average nuclear volume. (B) Bar graphs summarize the number per nuclei and the mean volume (±S.E.) of at least 500 Me3H3K9 foci analyzed. (C) The level of Ac3K9/14H3 expressed as the mean (±S.E.) of the fluorescence intensity of 100 nuclei analyzed for each treatment.

To determine whether cell migration involves spatial genome reorganization, we analyzed the distribution of all centromeres by fluorescent in situ hybridization (FISH) using a pan-centromeric DNA probe. By qualitative inspection of microscopic images, we observed that centromeres were distributed throughout the nucleus, but forming the typical aggregates composed by the centromeres of two or more chromosomes (chromocenters) (Figure 6). For quantitative analysis, we determined the volume occupied by these nuclear domains in control, anti-α_5_β_1 _and HGF/SF1-treated MLP29 cells. The mean volume of the chromocenters decreased drastically following α_5_β_1 _functional blockade (~6 μm^3^/chromocenter) or HGF/SF1 stimulation (~4 μm^3^/chromocenter) in comparison to untreated control cells (~8 μm^3^/chromocenter), while the average nuclear volume remained unchanged (~250 μm^3^) (Figures 7A).

To further explore the nuclear effects of α_5_β_1 _block or HGF/SF1 stimulation we analyzed the pattern of histone H3 trimethylated at lysine 9 (Me3K9H3) and acetylated at lysine 9 and 14 (Ac3K9/K14H3) by immunofluorescence analysis. Mouse cells have the majority of Me3K9H3 localized to prominent clusters of pericentromeric heterochromatin [14] and, consistent with previous reports, microscopic inspection of MLP29 cell preparations revealed that Me3K9H3 localized to chromocenters (Figure 6). However, detailed 3D analysis revealed that α_5_β_1 _functional blockade increased the average number of foci per nucleus, and this was concomitant with a decrease in their volume (Figure 7B). The changes in centromeres and Me3K9H3 foci spatial organization were not related to alterations in the nuclear volume due to technical artifact (mean nucleus volume ~250 μm^3^) or variation of the mean fluorescence intensity.

Since acetylated histone H3 exhibited a homogeneous nuclear distribution (Figure 6), rather than a focal pattern, we analyzed the levels of Ac3K9/K14H3 by flow cytometry and western blot, in addition to immunofluorescence, in order to determine the protein expression level and measure the mean intensity of Ac3K9/K14H3 more accurately. Interestingly, α_5_β_1 _functional blockade or HGF/SF1 stimulation significantly increased the levels of Ac3K9/K14H3 (Figure 7C; Additional files 8 and 9). Altogether, these observations indicate that alterations of the α_5_β_1_-mediated cell-ECM interactions during cell migration influence the overall spatial and functional organization of the nucleus.

### Positioning of genes encoding integrins during cell scattering

**Figure 8:**
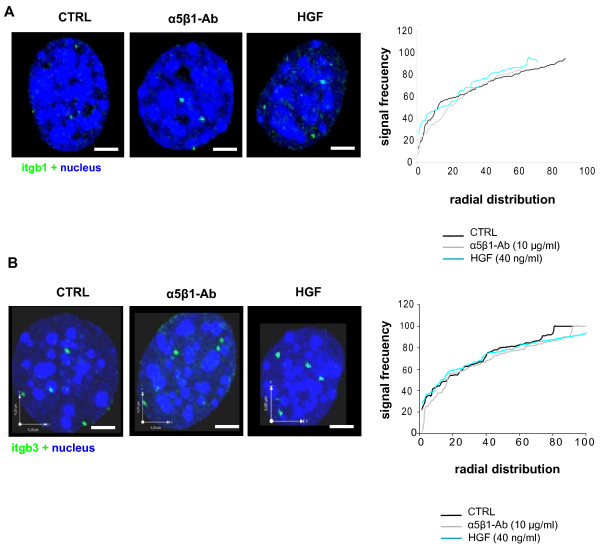
**Radial position of integrin genes during migration of MLP29 cells**. Three-dimensional FISH analysis was performed with BAC clones for the *Itgb1 *or *Itgb3 *loci and the position of fluorescent signals was determined using computational programs for image analysis. Left panels, 3D reconstruction of MLP29 cell nuclei subjected to FISH analysis with a probe for the *Itgb1 *and *Itgb3 *genes. Right panel, absolute radial position of these genes in control and treated cells. Scale bar = 3 μm.

Taking into account that α_5_β_1 _functional blockade and HGF/SF1 stimulation resulted in up-regulation of the genes encoding the integrin β_1 _and β_3 _sub-units; we asked whether these genes undergo repositioning. To this end, we analyzed the nuclear radial position of each gene independently by interphase 3D DNA-FISH and the results from 50-70 nuclei per experiment were plotted on cumulative distribution graphs. The preferential radial position of *Itgb1 *and *Itgb3 *was between 80% and 100% of the relative radius in control cells. Pair-wise comparisons by Kolmogorov-Smirnov statistical tests, demonstrated that these genes did not change their position upon α_5_β_1 _functional blockade or HGF/SF1 stimulation (p > 0, 05) (Figures 8A and 8B). Considering the increase in the expression of β_1 _and β_3 _integrins associated with cell scattering, these results suggest that the *Itgb1 *or *Itgb3 *radial positions do not depend on gene activity.

## Discussion

Invasive cell growth occurs not only during malignancy, but also takes place under physiological conditions during embryonic development and organ formation. In the present work, we demonstrate that functional blocking of α_5_β_1 _integrin induces cell dissociation and motility, and that this program involves changes in the structural and functional organization of the genome. Cell scattering induced by functional blockade of α_5_β_1 _integrin was statistically demonstrated by the wound healing assay, but the transwell assays did not show invasive capacity. Gerlitz et al. have reported that overexpression of the C-terminal domain of the histone H1E accelerated cell migration in the wound healing assays, but inhibited cell migration as measured by the transwell assay [15]. Likewise, our results for this test are contrasting, and this may be due to the fact that the experimental procedure includes the pre-treatment of the cells with the α_5_β_1 _functional blocking antibody, which may inhibit their adhesion to components of the transwell membrane and consequently cells can not invade. Alternatively, different migration mechanisms may be used in these two assays: In the wound healing assay, the cells use mesenchymal-like polarized motility and the transwell assay implies amoeboid-like movements [15]. Cell migration was accompanied by the activation of ERKs 1/2 and p38 MAPKs cell signaling pathways and a drastic increase in metabolic status. ERK 1/2 and p38 MAPKs phosphorylation occurs during migration of different cell types in response to various growth factors such as VEGF, EGF and TGF-β, and to components of the ECM [16]. Functional blockade of α_5_β_1 _in MLP29 cells, on the other hand, was able to up-regulate the expression of the hepatic stem/progenitor cell markers EpCAM, AFP and CK19, all shown to be associated with migration of epithelial and embryonic stem cells in vitro [17]. Furthermore, EpCAM participates in intercellular and cell-ECM interactions, and is expressed during liver regeneration [18].

Gene expression profiles documented here strongly support the notion that α_5_β_1 _integrin-mediated cell migration was associated with a larger number of differentially regulated genes than the process induced by HGF/SF1. Moreover, the number of up-regulated genes was higher than the number of down-regulated genes. These data fit very well with the results obtained from human fibroblasts which exhibited extensive down-regulation of genes when seeded onto low adhesion surfaces, which reduced cell scattering [19]. Following functional blockade of α_5_β_1 _integrin or stimulation with HGF/SF1, the most significantly up-regulated genes fell into the GO categories concerning response to external stimuli, biosynthesis and cell growth, and in particular genes involved in cell adhesion and migration. We also detected drastic changes in the expression of several members of the SWI/SNF family of chromatin remodeling complex. This consist of approximately ten ATP-dependent components and are thought to regulate genome function by altering the structure of chromatin [20].

One key question in cell biology is how cells reorganize their genome in response to mechanical conditioning. We detected that migration of MLP29 cells involved morphological changes, cytoskeleton reorganization, and nuclear architecture remodeling. The FISH analyses for the β_1_- and β_3_-integrin subunits revealed that the radial position of these genes within the interphase nucleus did not change. Meaburn and Misteli analyzed the radial distribution of four genes related to cell survival, mobility and migration, namely *AKT1*, *VEGF*, *ERBB2*, and *FGFR1 *in a cell-model of breast cancer. They found that, except for *VEGF*, the other loci underwent repositioning during tumorigenic differentiation [10]. However, it has also been shown that positional changes of chromosome territories only occur transiently at the beginning of the G1 phase, and then arrangements are stably maintained from mid G1 to early prophase during cell cycle [21], Our results are in agreement with this, and are supported by the flow cytometric analysis of DNA content which showed that MLP29 cells did not progress through the G1 phase of the cell cycle after α_5_β_1 _functional blockade. Furthermore, Harnicarova and collaborators have reported that nuclear arrangement of the *c-Myc *gene and its transcripts was conserved during enterocitic differentiation of HT-29 cells [22]. In contrast to this conserved spatial positioning of β_1_- and β_3_-integrin gene loci, FISH analysis with a pan-centromeric probe revealed that the average number of chromocenters per nucleus increased, while their mean volume decreased following α_5_β_1 _functional blockade and HGF/SF1 stimulation, indicating that the spatial distribution of individual centromeres changes in response to the forces generated during cell migration. In close agreement with these results, a study on the position of centromeres of human chromosomes 3, 11 and 16 showed that the distance between centromeres decreases when fibroblasts are seeded onto matrices resembling 3D structures, compared to flat 2D surfaces, which generate different tensile forces and activate distinct signaling cascades [19]. We also found that during MLP29 cell migration, the volume of Me3K9H3 foci decreased concomitantly with an increase in their average number. These findings, although expected because Me3K9H3 localizes preferentially to pericentromeric heterochromatin, corroborate the data obtained by FISH with pan-centromeric probes demonstrating chromocenter disaggregation during cell scattering. It has been shown that progressive chromocenter clustering occurs during cell differentiation and that this is associated with increasing levels of DNA methylation [23]. Our immunostaining assays with antibodies against Me3K9H3 showed that the nuclear fluorescent intensity level was the same after functional blockade of α_5_β_1_, suggesting that cell migration may occur without changes in the methylation status of histone H3. Most important, these experiments demonstrated that the architectural changes of chromocenters associated with this program are completely opposite to those leading to terminally differentiated or quiescent cells. Thus, it appears that cell scattering involves significant spatial reorganization of the heterochromatic blocks of the genome associated with substantial changes in the expression level of genes encoding for chromatin remodeling factors.

## Conclusion

We provide evidence that the functional blockade of α_5_β_1 _integrin in hepatic progenitor cells induces cell scattering and cytoskeleton reorganization in a manner similar to stimulation with HGF/SF1. This process is accompanied by activation of the MAPK pathway and an increase in the expression levels of the early HCC markers, Ep-CAM, AFP and CK19. Gene expression arrays showed a massive change in the expression of chromatin remodeling and transcription factors. These functional changes of hepatic progenitor cells are associated with conspicuous structural reorganization of the cell nucleus.

## Methods

### Cell Culture and treatments

Mouse hepatic progenitor MLP29 and mouse HCC Hep16 cells were maintained in RPMI 1640 medium supplemented with 10% Fetal Bovine Serum (FBS) at 37°C in a humidified 5% CO_2 _atmosphere. For experiments, after cell plating, the old medium was replaced with fresh medium containing low FBS (0.1%) and the cells were maintained under this serum starvation condition for at least 16 hours. Then the cells were treated with 10 μg/ml of a specific function-blocking anti-α_5_β_1 _integrin antibody (clone BMB5) purchased from Chemicon-Millipore (Temecula, CA, USA) or 20 ng/ml of HGF/SF1. When appropriate, 20 μM of U0126 (ERK inhibitor) was used for 24 hours. Control experiments were carried out by adding equal amounts of DMSO, ethanol or an IgG control Ab.

### Antibodies and reagents

MEK1 and MEK2 specific inhibitor U0126 was purchased from Calbiochem (San Diego, CA), dissolved in DMSO, and stored in the dark as a 10 mM stock solution at -20°C. The components of the ECM, fibronectin (FN) and vitronectin (VN) were purchased from Sigma (St Louis, MO), laminin (LMN) was purchased from Chemicon (Temecula, CA) and collagen type I (COL I) was purchased from BD Biosciences-Europe (Erembodegem, Belgium). hrHGF was purchased from Peprotech (Rocky Hills, NJ). Specific antibodies against β_1 _and β_3 _integrin subunits, CK19, Acetyl histone H3 (Lys9/14), and tri-Methyl histone H3 were purchased from Chemicon-Millipore (Temecula, CA), and E-Cadherin antibody was from R&D systems. Phospho p42/p44 MAPKinase antibody (Thr 202/Tyr204), total MAPKinase; phospho PKB/Akt (Ser 473) and total PKB/Akt antibodies were purchased from Cell Signaling Biotechnologies (Beverly, MA, USA). β-actin, Ep-CAM and AFP antibodies were purchased from Santa Cruz Biotechnology (Santa Cruz, CA, USA). Pan-centromeric STAR-FISH™ probe were purchased from Cambio (Cambridge, UK).

### Immunofluorescence

Sub-cellular localization of β_1 _integrins, Ac3K9/k14H3, Me3K9H3 and phospho-ERKs was assessed by immunofluorescence. Briefly, the cells were fixed in 4% paraformaldehyde (w/v) in PBS, rinsed in TBS and then permeabilized in 0.5% TBS-Triton X-100 (T-TBS 0.5%) for 10 minutes at RT. After blocking with 2% BSA in TBS for 10 min at RT, the cells were incubated with the primary antibody for 1 hour at RT. The detection was performed with secondary antibodies conjugated to FITC or Texas Red. To study the actin cytoskeleton and morphological changes, the cells were stained with phalloidin conjugated to Texas-red. After exhaustive washings with cold TBS, the slides were mounted with Vectashield containing DAPI (Vector Laboratories, CA, USA). Images were acquired with an Olympus BX61 epifluorescence microscope or a Leica DMIRE-2 confocal microscope.

### Flow cytometric evaluation of RGD-binding integrins

MLP29 hepatic progenitor and Hep16 HCC cells were seeded and allowed to attach for 24 hours. Cells were then harvested by scraping, subsequently counted, and then incubated with antibodies against the α_5_β_1 _heterodimer and β_1 _integrin subunit (3 μg/500 × 10^3 ^cells/100 μl) for 1 hour at 4°C. After that, cells were washed twice with cold PBS containing 1% FBS, and incubated with a CY2-conjugated secondary antibody for 30 minutes at 4°C. Cells were then analyzed in a FACScanto II flow cytometer (Becton Dickinson). FACS Diva software (Becton Dickinson) was used for data acquisition and analysis.

### Flow cytometric analysis of Ac3K9/K14H3 and DNA content

MLP29 hepatic progenitor cells were treated with the α_5_β_1 _blocking antibody or HGF/SF1 for 6 hours, and then the cells were harvested. For Ac3K9/K14H3 detection, cells were fixed in 1% paraformaldehyde for 15 minutes and stored in 70% ethanol at -20°C. After fixation, cells were permeabilized with PBS-0.25% Triton X-100 for 10 minutes, washed with cold PBS-1% FBS and incubated with an anti-Ac3K9/K14H3 antibody for 2 hours at room temperature. Then, the samples were washed twice and incubated with a FITC-conjugated secondary antibody. For DNA staining, the cells were washed again and treated with RNAse A (0.2 mg/ml) in PBS at 37°C for 20 minutes. Propidium Iodide (20 μg/ml) was added to the cell suspension, and incubated 30 minutes at RT protected from light. FACSDiva software (Becton Dickinson) was used for data acquisition and analysis.

### Cell adhesion studies

Cell adhesion was assessed by the MTT assay using the CellTiter 96^® ^Non-Radioactive Cell Proliferation assay (Promega Corporation, WI). Briefly, MLP29 and Hep16 cells were seeded at 50 × 10^3 ^cells/well in 96-well plates coated with FN (10 μg/ml), VN (1 μg/ml), LMN (10 μg/ml), COL I (0.05 mg/ml) and BSA (0.2 mg/ml) as a control, and allowed to attach for 1 hour. After extensive washings, the medium was aspirated carefully and the dye solution added. After 3-5 hours of incubation, the reaction was stopped with the "Solubilization/Stop" buffer provided with the kit. Following overnight incubation at 37°C, absorbance at 570 nm was determined with a SpectraMax M2 plate reader (Molecular Devices, Sunnyvale, CA, USA).

### Cell migration and cell invasion assays

To study cell migration we performed the wound healing assay. MLP29 and Hep16 cells were plated seeded on six wells plastic tissue culture dishes and grown to confluence. Then, the cell monolayer was scratched with a p200 pipette tip, washed twice, and incubated in growth medium with 0.1% FBS containing 10 μg/ml of α_5_β_1 _functional-blocking antibody or 40 ng/ml of HGF/SF1 for 20 hours. Microscope images were taken from at least three fields for each experiment. The distance covered by the migrating cells was calculated as the mean of six different measurements along the scratch using the Gimp 2.0 image software.

To assess cell invasion, we used the QCM™ 24 wells Cell Invasion Assay from Chemicon-Millipore (Temecula, CA, USA) according to manufacturer's instructions. Briefly, 500 × 10^3 ^MLP29 or Hep16 serum starved cells were loaded into an insert containing an eight μm pore size and polycarbonate membrane coated with ECMatrix™ in the presence of α_5_β_1 _functional-blocking antibody or HGF/SF1 at the concentrations stated above and incubated for 48 and 72 hours. Invaded cells at the bottom of the membrane were detached, lysed and detected by the CyQUANT™ GR Dye in a SpectraMax M2 plate reader (Molecular Devices, Sunnyvale, CA, USA).

### Immunoblotting analysis of ERK1/ERK2 MAPK, p-ERK1-ERK2 MAPK, AKT, pAKT, integrin sub-units and histone acetylation

Cells were cultured in a serum free medium for 16-18 hours and then stimulated with HGF/SF1 or treated with α_5_β_1 _functional-blocking antibodies for up to 24 hours. After treatment, cells were harvested, washed twice in PBS, and lysed with the Cell Lysis Buffer (Cell Signaling Biotechnologies) and 1 mM phenylmethylsulfonylfluoride for 30 minutes on ice. The lysates were then cleared by centrifugation (14000 rpm for 15 minutes at 4°C). Protein concentration was determined using the Pierce protein assay kit (Rockford, IL, USA). For analysis of ERK1/2 MAPKs and Akt, equal amounts of protein (20 μg) were resuspended in 5× Laemli buffer (10 minutes at 100°C), resolved by electrophoresis on a 10% SDS-PAGE, and transferred onto nitrocellulose membranes (Amersham Biosciences). For analysis of histone H3 acetylation, 50 μg of protein was resolved on a 15% SDS-PAGE and transferred onto a nitrocellulose membrane. The membranes were then blocked with TBS-T (25 mM Tris-HCl, 150 mM NaCl (pH 7.5), and 0.05% Tween 20) containing 5% (w/v) non-fat dry milk and incubated overnight at 4°C with specific antibodies. After incubation, the membranes were washed in TBS-T, and HRP-conjugated anti-rabbit, anti-mouse or anti-goat antibodies were added for 1 hour as secondary antibodies. Immunodetection was performed using the Western Lighting chemiluminescence reagent from Perkin Elmer (Boston, MA, USA).

### Fluorescent in Situ Hybridization (FISH)

Cells were fixed following protocols designed for the preservation of the three dimensional structure of the nuclei. For centromere analysis, the cells were fixed in 4% paraformaldehyde in PBS 0.3× (w/v) for 10 minutes and then permeabilized in PBS/Triton X-100 0.5% for 20 minutes at RT and transferred to 20% Glycerol/PBS. After 1 hour, the cells were further permeabilized by freezing and thawing cycles in liquid nitrogen. The probe was dissolved in 50%formamide/20% dextran-sulfate, denatured and incubated along with the denatured cell-targets in a humid chamber at 37°C overnight. For locus-specific 3D FISH, the fixation of the cells was performed according to the protocol described previously [8], with slight modifications. Briefly, the coverslips were fixed for 10 minutes in 4% paraformaldehyde in PBS (w/v). After permeabilization in 0.5% saponin (w/v) PBS/Triton X-100 0.5% (v/v) for 30 minutes, the slides were washed in PBS for 2 minutes at RT and treated with 0.1 N HCl for 20 minutes. Next, the cells were washed with PBS and 2 × SSC for 2 minutes, treated with RNAse A, transferred to 2 × SSC/50% formamide 0.1% NaAz and stored at 4°C. We used probes prepared with the BAC clones RP23-303A11 y RP23-343N12 specific for the *Itgb1 *or the *Itgb3 *genes, respectively. The probes were labeled by nick translation with dUTP conjugated with biotin (Roche) or digoxigenin (Roche). Before hybridization, the probes were pre-denatured at 80°C for 5 minutes, then denatured along with the target DNA at 75°C for 5 minutes and incubated at 37°C for 72 hours in a humified chamber. For the post-hybridization washings, the coverslips were immersed in 50% formamide/2 × SSC, 1 × SSC, and 4 × SSC/tween-20 0.1% (v/v) (4T) each for 5 minutes at 47°C. Following one more wash with 4T at RT, the coverslips were blocked with 3% BSA in 4T (wt/v), washed in 4T at RT for 5 minutes and incubated for 1 h with antibodies against biotin or digoxigenin in blocking solution. Then the coverslips were incubated with the respective secondary antibodies for 1 hour, washed twice with PBS for 5 minutes and mounted with Vectashield containing DAPI (Vector Laboratories, CA, USA).

### Image acquisition and analysis

Stacks of images scanning the whole nucleus were acquired with an axial separation of 250 nm using a laser-scanning microscope Leica DM IRE2 (Leica Microsystems Heidelberg GmbH). For quantitative analysis of chromocenters and Me3K9H3 foci, regions of interest (ROI) including each nucleus were directly generated on the z-stack using the Volocity.4.3^® ^software (Improvision, Image, Processing and Vision Company Limited) and then the intensity level, volume and center of mass of all objects contained within each ROI was automatically recorded. Between 500-1000 elements (chromocenters or Me3K9H3 foci) per treatment were analyzed. For statistical analysis, the "proportions test" was used to compare the average number of elements per nucleus for each treatment. For the comparison of mean volumes and mean fluorescence intensity, we applied the "t-student" test. Fluorescence intensity of Ac3K9/K14H3 was directly measured on the Z-stack using the Volocity.4.3^® ^software (Improvision, Image, Processing and Vision Company Limited).

To determine the 3D radial position of any given signal, the shortest distance from the center of mass of the nucleus to the periphery, which included the center of mass of the fluorescent signal, was directly measured on the z-stack using the Volocity.4.3^® ^software. The absolute distances from the nucleus center to the gene were normalized as a fraction of nuclear radius, to account for natural variations in nuclear size that may influence positioning. Cell nuclei were subdivided into five concentric shells each corresponding to 20% of the nuclear radius, and the radial positioning data was binned into these five sub-domains. Graphs were made using Microsoft Excel Software. For quantitative measurements, 50-70 nuclei from multiple experiments were analyzed. Statistical differences (p < 0.05) between the distributions of a gene in different conditions were determined using the 1D Kolmogorov-Smirnov test.

### Microarray data acquisition and analysis

RNA was isolated from cell plates subjected to the different treatments using the RNAeasy Mini kit (Qiagen) and treated with RNAase-free DNAase (Qiagen). The cDNA was synthesized from 1 μg of RNA using the Reverse Transcriptase kit (Promega) following the manufacturer's recommendations. Expression profiling was performed using the Affimetrix GeneChip ^® ^technology, following the protocols recommended by the manufacturers. Affymetrix raw files (.cel, Mouse430A_2) obtained from untreated control cells, treated with anti-α_5_β_1 _antibodies, and stimulated with HGF/SF1 were collectively analyzed by using the R package "AFFYLMGUI" (http://www.bioconductor.org) [24]. Data were background corrected by the rma method [25]. The p-values obtained by testing for differentially expressed genes were calculated using "AFFYLMGUI" and exported as text. Extraction of GO terms for all genes on the Mouse430A_2 chips was used for functional analysis. Hypergeometric p-values for testing over representation of genes in GO categories were calculated as described in Masseroli et al., and the p-values were corrected for multiple testing by the Holm method [26,27]. The gene set enrichment analysis was done as described previously [28,29]. The permutation statistics for the heat-plots were calculated for each gene set. This was done by considering the expression level for all genes of each set defined by Molecular Signatures Database. The p-values for the heat-plots were calculated as n_down_/N for down-regulated genes and as 1-n_up_/N for up-regulated genes, where n_down _is the number of times a permutated data set resulted in a down regulation that was stronger or equal to the one observed for the unpermutated data. n_up _was calculated in a similar way for up-regulated genes. N was the total number of permutation which was set to 10000. To assess if a specific gene set was up- or down-regulated for a specific sample class the expression values from a specific class were compared with the expression values in all other sample classes [30]. The calculation of 1-n_up_/N for up-regulated gene sets allows co-plotting both up and down-regulated gene sets in the same heat-diagram. Mouse genes were mapped to human genes by using HomologGene available at NCBI. Microarray data is publicly available at http://www.ncbi.nlm.nih.gov/geo/query/acc.cgi?acc=GSE23853, Gene Expression Omnibus (GEO) accession number GSE23853.

## Authors' contributions

LV performed the cell experiments, image analysis and drafted the manuscript. FR, JT-F participated in statistical and image analyses. RM performed the microarray-data statistical analysis. AL assisted with cell experiments. LP conceived and coordinated the study, performed the FISH experiments and wrote the manuscript. All authors read and approved the final manuscript.

## Supplementary Material

Additional file 1 **MLP29 cells were treated with a specific functional blocking antibody against α_5_β_1 _integrin or stimulated with HGF/SF1 for 24 hours**. Then immunofluorescent detection of invasiveness and hepatic stem cell (HSC) biomarkers showed that cell migration was associated with an increase in the expression levels of β_1 _and β_3 _integrin sub-units. The treatments also increased the expression of the HSC markers Ep-CAM, AFP and CK19, all related to the invasive growth of epithelial cells. Click here for file

Additional file 2 **Following treatment with the α_5_β_1 _blocking antibody, viability of the cells was determined using the MTT reduction assay**. The effects of exposure of the cells to the α_5_β_1 _specific antibody or stimulation with HGF/SF1 were analyzed by generating concentration-effect curves as a plot of the fold-time increase in the fraction of surviving cells *versus *antibody concentration. The data presented summarize the mean (±S.D.) of three independent experiments each performed in triplicate. Click here for file

Additional file 3 **Flow cytometric analysis of the DNA content of hepatic progenitor cells was performed following treatment with an α_5_β_1_-specific antibody in the presence or absence of the MEK inhibitor U0126**. α_5_β_1 _inhibition slightly increased (statistically non-significant) the cell sub-population in the S phase of the cell cycle and this is concomitant with a decrease in the percentage of cells in the G0/G1 sub-compartment of the cell cycle. These effects on the S-phase were not reversed upon inhibition of the proliferative MAPK pathway. Click here for file

Additional file 4 **Flow cytometric analysis of the DNA content of hepatic progenitor cells was performed following treatment with an α_5_β_1_-specific antibody in the presence or absence of the MEK inhibitor U0126**. The percentages of cells in each sub-compartment of the cell cycle are plotted as a function of the different treatments. One experiment, representative of three independent experiments, is shown. Click here for file

Additional file 5 **Frequencies of up- or down-regulated genes in the three main GO categories: Cellular Components (CC), Biological Processes (BP) and Molecular Function (MF)**. Click here for file

Additional file 6 **Most significantly up-regulated genes within the biological processes and cellular components GO categories in MLP29 cells upon α_5_β_1 _integrin functional blockade**. Click here for file

Additional file 7 **Most significantly down-regulated genes in MLP29 cells upon α_5_β_1 _integrin functional blockade**. Click here for file

Additional file 8 **Flow cytometric and immunoblot analyses of Ac3K9/14 H3 in the context of DNA content of MLP29 cells treated with an anti-α_5_β_1 _specific antibody**. Bivariate cytograms indicate the levels of expression of Ac3K9/14 H3 in each sub-compartment of the cell cycle. Bar graphs show the percentage of FITC-positive cells. The data presented are representative of three independent experiments. For immunoblot analysis, treated MLP29 cells were lysed and total protein (50 μg) was resolved by SDS-PAGE and analyzed by immunoblot for Ac3K9/14 H3. Click here for file

Additional file 9 **Flow cytometric and immunoblot analyses of Ac3K9/14 H3 in the context of DNA content of MLP29 cells treated with HGF/SF1**. Bivariate cytograms indicate the levels of expression of Ac3K9/14 H3 in each sub-compartment of the cell cycle. Bar graphs show the percentage of FITC-positive cells. The data presented are representative of three independent experiments. For immunoblot analysis, treated MLP29 cells were lysed and total protein (50 μg) was resolved by SDS-PAGE and analyzed by immunoblot for Ac3K9/14 H3. Click here for file

## References

[B1] Boccaccio C Comoglio PM Invasive growth: a MET-driven genetic programme for cancer and stem cells Nat Rev Cancer 2006 6 8 637 645 10.1038/nrc1912 16862193

[B2] Gentile A Trusolino L Comoglio PM The Met tyrosine kinase receptor in development and cancer Cancer Metastasis Rev 2008 27 1 85 94 10.1007/s10555-007-9107-6 18175071

[B3] Chan PC Chen SY Chen CH Chen HC Crosstalk between hepatocyte growth factor and integrin signaling pathways J Biomed Sci 2006 13 2 215 223 10.1007/s11373-005-9061-7 16496226

[B4] Streuli CH Integrins and cell-fate determination J Cell Sci 2009 122 Pt 2 171 177 10.1242/jcs.018945 19118209 PMC2714415

[B5] Bertotti A Comoglio PM Trusolino L Beta4 integrin is a transforming molecule that unleashes Met tyrosine kinase tumorigenesis Cancer Res 2005 65 23 10674 10679 10.1158/0008-5472.CAN-05-2827 16322210

[B6] Trusolino L Serini G Cecchini G Besati C Ambesi-Impiombato FS Marchisio PC De Filippi R Growth factor-dependent activation of alphavbeta3 integrin in normal epithelial cells: implications for tumor invasion J Cell Biol 1998 142 4 1145 1156 10.1083/jcb.142.4.1145 9722624 PMC2132885

[B7] Parada LA Sotiriou S Misteli T Spatial genome organization Exp Cell Res 2004 296 1 64 70 10.1016/j.yexcr.2004.03.013 15120995

[B8] Royo F Paz N Espinosa L McQueen PG Vellon L Parada LA Spatial link between nucleoli and expression of the Zac1 gene Chromosoma 2009 118 6 711 722 10.1007/s00412-009-0229-1 19649645 PMC2783200

[B9] Wiblin AE Cui W Clark AJ Bickmore WA Distinctive nuclear organisation of centromeres and regions involved in pluripotency in human embryonic stem cells J Cell Sci 2005 118 Pt 17 3861 3868 10.1242/jcs.02500 16105879

[B10] Meaburn KJ Misteli T Locus-specific and activity-independent gene repositioning during early tumorigenesis J Cell Biol 2008 180 1 39 50 10.1083/jcb.200708204 18195100 PMC2213600

[B11] Medico E Mongiovi AM Huff J Jelinek MA Follenzi A Gaudino G Parsons JT Comoglio PM The tyrosine kinase receptors Ron and Sea control "scattering" and morphogenesis of liver progenitor cells in vitro Mol Biol Cell 1996 7 4 495 504 8730094 10.1091/mbc.7.4.495 PMC275904

[B12] Ingber DE Tensegrity II. How structural networks influence cellular information processing networks J Cell Sci 2003 116 Pt 8 1397 1408 10.1242/jcs.00360 12640025

[B13] Alison MR Murphy G Leedham S Stem cells and cancer: a deadly mix Cell Tissue Res 2008 331 1 109 124 10.1007/s00441-007-0510-7 17938965

[B14] Wu R Terry AV Singh PB Gilbert DM Differential subnuclear localization and replication timing of histone H3 lysine 9 methylation states Mol Biol Cell 2005 16 6 2872 2881 10.1091/mbc.E04-11-0997 15788566 PMC1142431

[B15] Gerlitz G Livnat I Ziv C Yarden O Bustin M Reiner O Migration cues induce chromatin alterations Traffic 2007 8 11 1521 1529 10.1111/j.1600-0854.2007.00638.x 17822403

[B16] Huang C Jacobson K Schaller MD MAP kinases and cell migration J Cell Sci 2004 117 Pt 20 4619 4628 10.1242/jcs.01481 15371522

[B17] Kirkland SC Ying H Alpha2beta1 integrin regulates lineage commitment in multipotent human colorectal cancer cells J Biol Chem 2008 283 41 27612 27619 10.1074/jbc.M802932200 18664572 PMC2562061

[B18] Trzpis M Popa ER McLaughlin PM van Goor H Timmer A Bosman GW de Leij LM Harmsen MC Spatial and temporal expression patterns of the epithelial cell adhesion molecule (EpCAM/EGP-2) in developing and adult kidneys Nephron Exp Nephrol 2007 107 4 e119 131 10.1159/000111039 18025791

[B19] Dalby MJ Gadegaard N Herzyk P Sutherland D Agheli H Wilkinson CD Curtis AS Nanomechanotransduction and interphase nuclear organization influence on genomic control J Cell Biochem 2007 102 5 1234 1244 10.1002/jcb.21354 17427951

[B20] Smith CL Peterson CL A conserved Swi2/Snf2 ATPase motif couples ATP hydrolysis to chromatin remodeling Mol Cell Biol 2005 25 14 5880 5892 10.1128/MCB.25.14.5880-5892.2005 15988005 PMC1168809

[B21] Walter J Schermelleh L Cremer M Tashiro S Cremer T Chromosome order in HeLa cells changes during mitosis and early G1, but is stably maintained during subsequent interphase stages J Cell Biol 2003 160 5 685 697 10.1083/jcb.200211103 12604593 PMC2173351

[B22] Harnicarova A Kozubek S Pachernik J Krejci J Bartova E Distinct nuclear arrangement of active and inactive c-myc genes in control and differentiated colon carcinoma cells Exp Cell Res 2006 312 20 4019 4035 10.1016/j.yexcr.2006.09.007 17046748

[B23] Brero A Easwaran HP Nowak D Grunewald I Cremer T Leonhardt H Cardoso MC Methyl CpG-binding proteins induce large-scale chromatin reorganization during terminal differentiation J Cell Biol 2005 169 5 733 743 10.1083/jcb.200502062 15939760 PMC2171616

[B24] Wettenhall JM Simpson KM Satterley K Smyth GK affylmGUI: a graphical user interface for linear modeling of single channel microarray data Bioinformatics 2006 22 7 897 899 10.1093/bioinformatics/btl025 16455752

[B25] Bolstad BM Irizarry RA Astrand M Speed TP A comparison of normalization methods for high density oligonucleotide array data based on variance and bias Bioinformatics 2003 19 2 185 193 10.1093/bioinformatics/19.2.185 12538238

[B26] Masseroli M Martucci D Pinciroli F GFINDer: Genome Function INtegrated Discoverer through dynamic annotation, statistical analysis, and mining Nucleic Acids Res 2004 32 Web Server W293 300 10.1093/nar/gkh432 15215397 PMC441570

[B27] Holm S A simple sequentially rejective multiple test procedure Scand J Stat 1979 6 65 70

[B28] Mootha VK Lindgren CM Eriksson KF Subramanian A Sihag S Lehar J Puigserver P Carlsson E Ridderstrale M Laurila E PGC-1alpha-responsive genes involved in oxidative phosphorylation are coordinately downregulated in human diabetes Nat Genet 2003 34 3 267 273 10.1038/ng1180 12808457

[B29] Virtaneva K Wright FA Tanner SM Yuan B Lemon WJ Caligiuri MA Bloomfield CD de La Chapelle A Krahe R Expression profiling reveals fundamental biological differences in acute myeloid leukemia with isolated trisomy 8 and normal cytogenetics Proc Natl Acad Sci USA 2001 98 3 1124 1129 10.1073/pnas.98.3.1124 11158605 PMC14719

[B30] Subramanian A Tamayo P Mootha VK Mukherjee S Ebert BL Gillette MA Paulovich A Pomeroy SL Golub TR Lander ES Gene set enrichment analysis: a knowledge-based approach for interpreting genome-wide expression profiles Proc Natl Acad Sci USA 2005 102 43 15545 15550 10.1073/pnas.0506580102 16199517 PMC1239896

