# Supplementary figures and images for: Functional blockade of α5β1 integrin induces scattering and genomic landscape remodeling of hepatic progenitor cells
Source: BMC Cell Biol. 2010 Oct 19;11:81. doi: 10.1186/1471-2121-11-81 (PMC2967514; doi:10.1186/1471-2121-11-81)

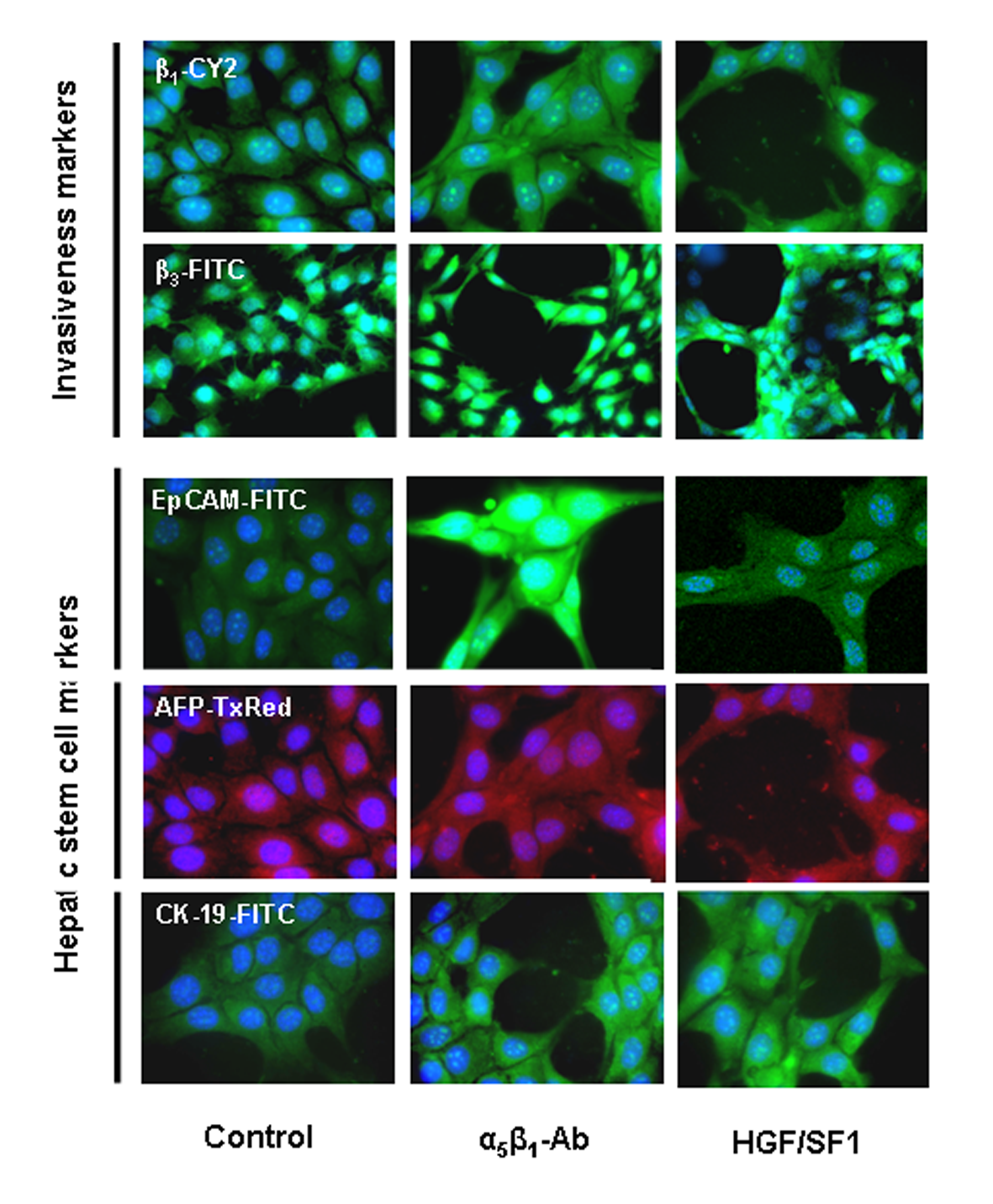

Supplement: Additional file 1 — MLP29 cells were treated with a specific functional blocking antibody against α5β1 integrin or stimulated with HGF/SF1 for 24 hours. Then immunofluorescent detection of invasiveness and hepatic stem cell (HSC) biomarkers showed that cell migration was associated with an increase in the expression levels of β1 and β3 integrin sub-units. The treatments also increased the expression of the HSC markers Ep-CAM, AFP and CK19, all related to the invasive growth of epithelial cells. [file 1471-2121-11-81-S1.TIFF]

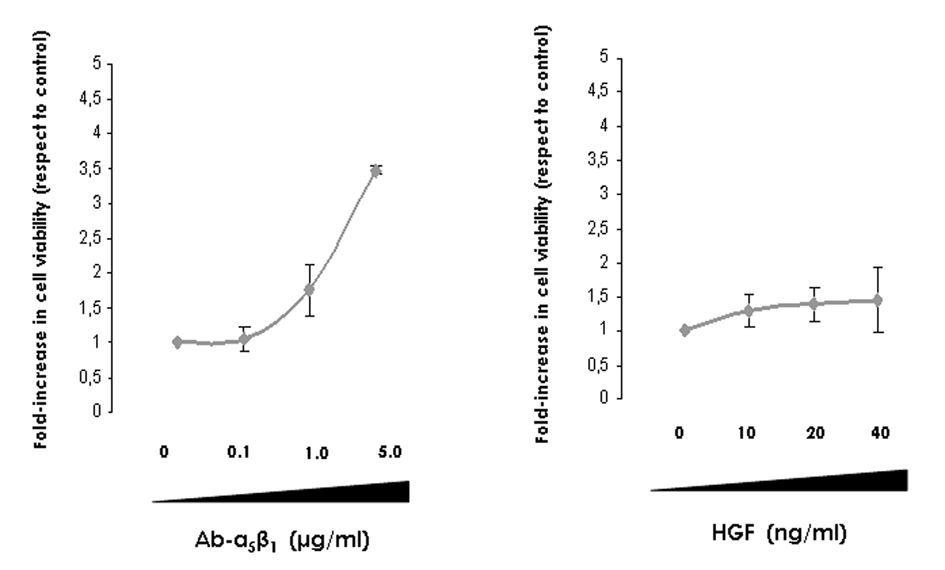

Supplement: Additional file 2 — Following treatment with the α5β1 blocking antibody, viability of the cells was determined using the MTT reduction assay. The effects of exposure of the cells to the α5β1 specific antibody or stimulation with HGF/SF1 were analyzed by generating concentration-effect curves as a plot of the fold-time increase in the fraction of surviving cells versus antibody concentration. The data presented summarize the mean (±S.D.) of three independent experiments each performed in triplicate. [file 1471-2121-11-81-S2.TIFF]

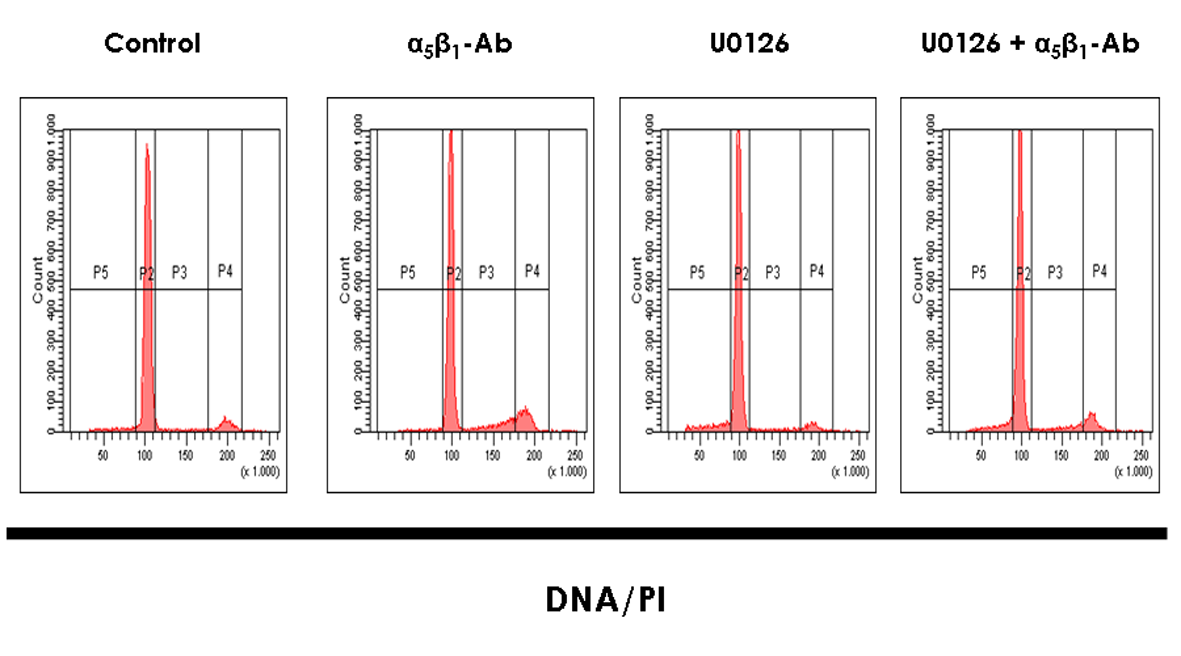

Supplement: Additional file 3 — Flow cytometric analysis of the DNA content of hepatic progenitor cells was performed following treatment with an α5β1-specific antibody in the presence or absence of the MEK inhibitor U0126. α5β1 inhibition slightly increased (statistically non-significant) the cell sub-population in the S phase of the cell cycle and this is concomitant with a decrease in the percentage of cells in the G0/G1 sub-compartment of the cell cycle. These effects on the S-phase were not reversed upon inhibition of the proliferative MAPK pathway. [file 1471-2121-11-81-S3.TIFF]

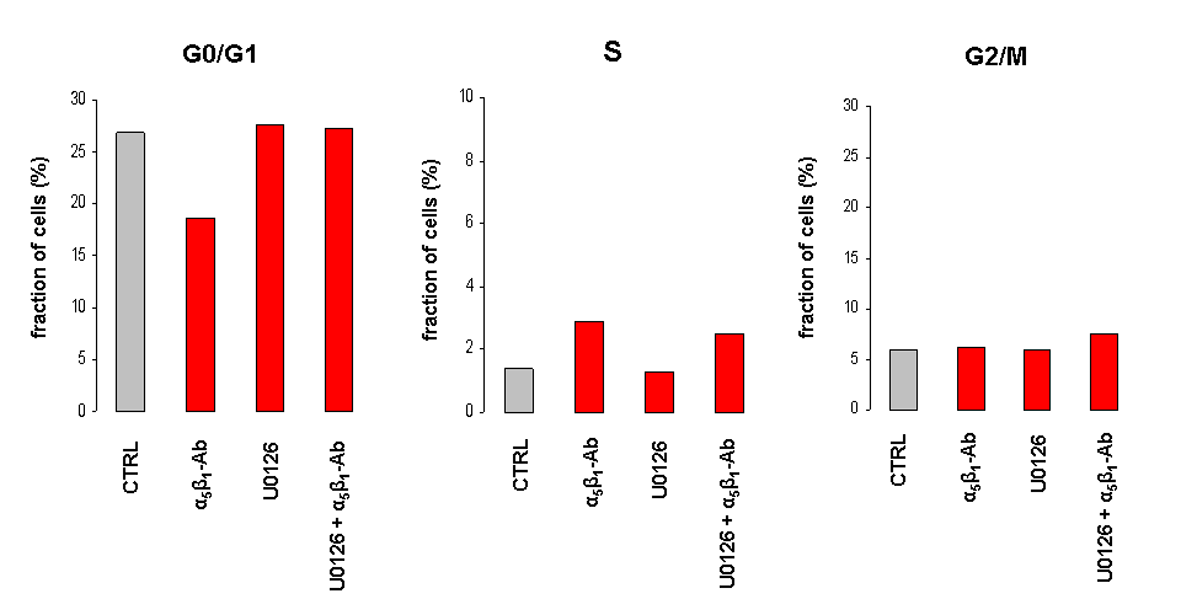

Supplement: Additional file 4 — Flow cytometric analysis of the DNA content of hepatic progenitor cells was performed following treatment with an α5β1-specific antibody in the presence or absence of the MEK inhibitor U0126. The percentages of cells in each sub-compartment of the cell cycle are plotted as a function of the different treatments. One experiment, representative of three independent experiments, is shown. [file 1471-2121-11-81-S4.TIFF]

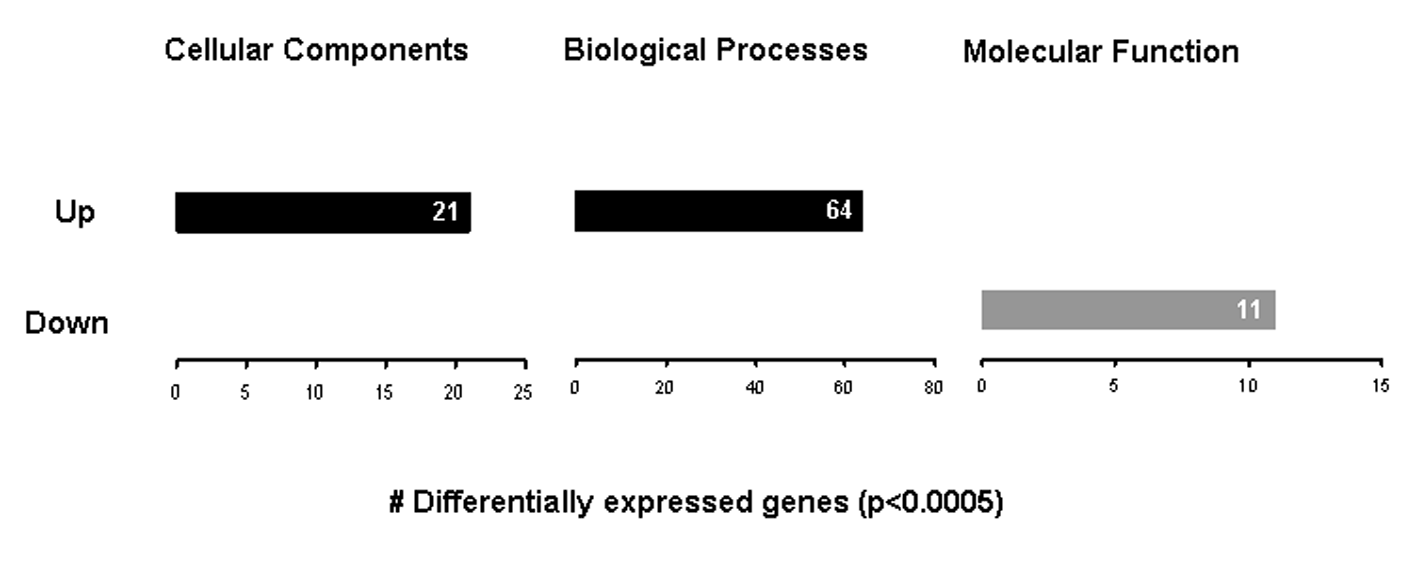

Supplement: Additional file 5 — Frequencies of up- or down-regulated genes in the three main GO categories: Cellular Components (CC), Biological Processes (BP) and Molecular Function (MF). [file 1471-2121-11-81-S5.TIFF]

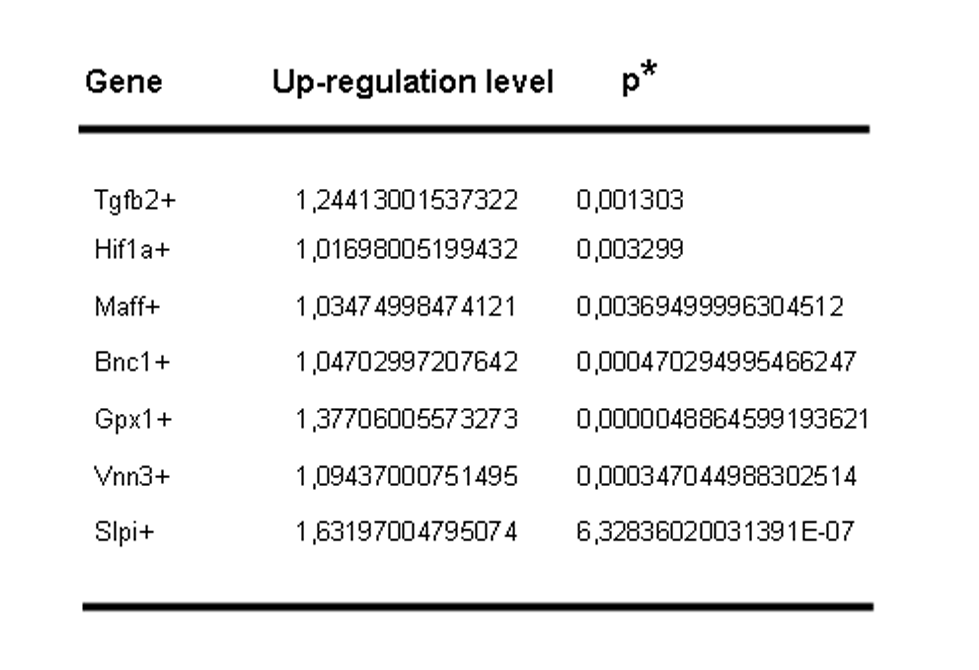

Supplement: Additional file 6 — Most significantly up-regulated genes within the biological processes and cellular components GO categories in MLP29 cells upon α5β1 integrin functional blockade. [file 1471-2121-11-81-S6.TIFF]

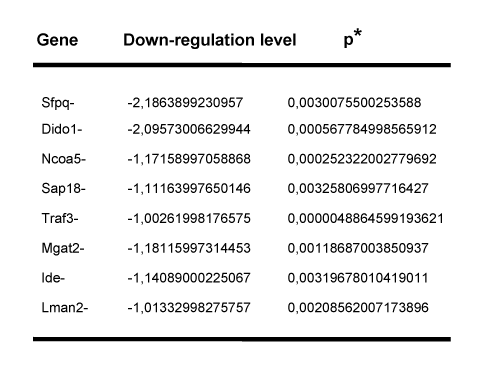

Supplement: Additional file 7 — Most significantly down-regulated genes in MLP29 cells upon α5β1 integrin functional blockade. [file 1471-2121-11-81-S7.TIFF]

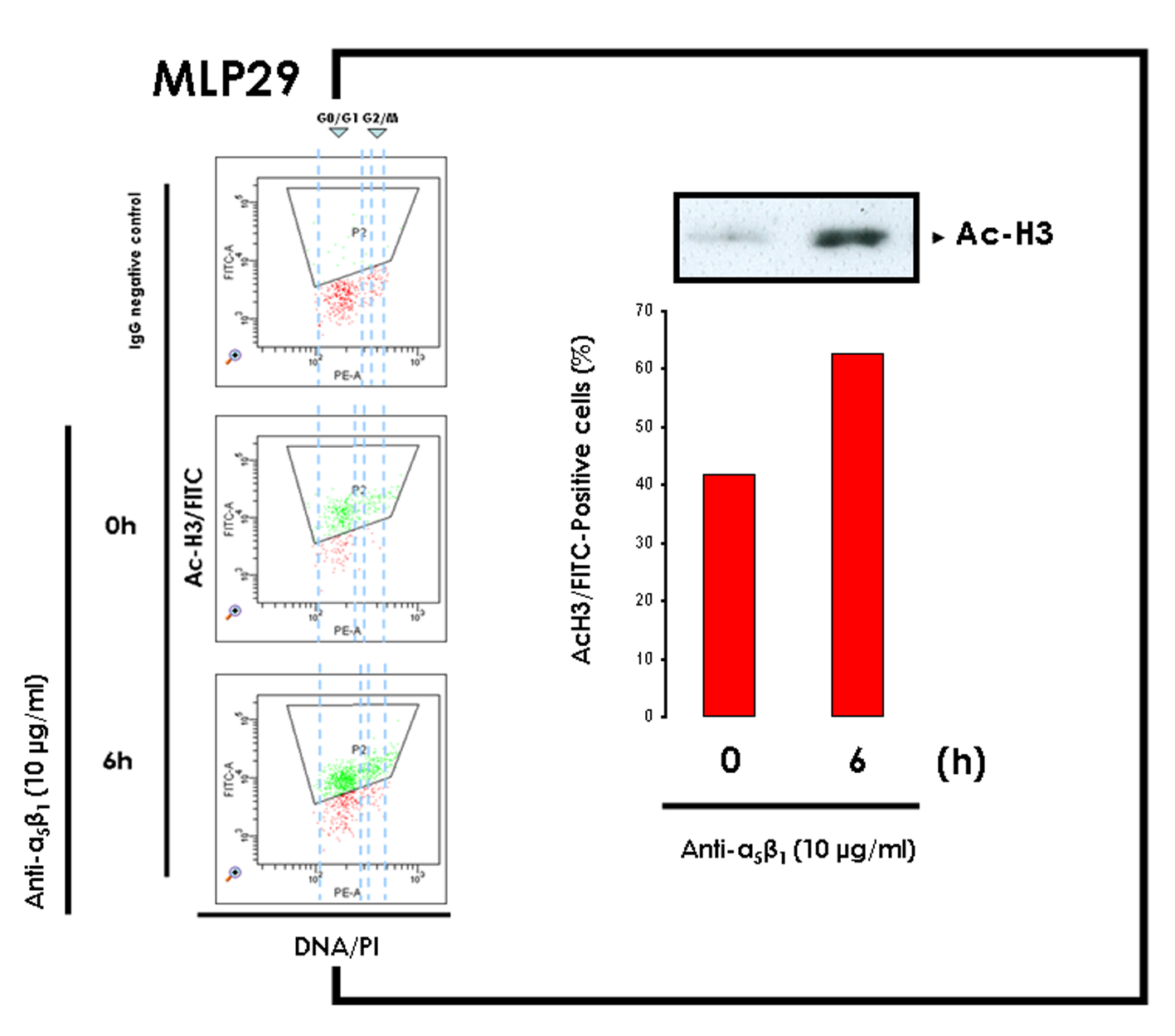

Supplement: Additional file 8 — Flow cytometric and immunoblot analyses of Ac3K9/14 H3 in the context of DNA content of MLP29 cells treated with an anti-α5β1 specific antibody. Bivariate cytograms indicate the levels of expression of Ac3K9/14 H3 in each sub-compartment of the cell cycle. Bar graphs show the percentage of FITC-positive cells. The data presented are representative of three independent experiments. For immunoblot analysis, treated MLP29 cells were lysed and total protein (50 μg) was resolved by SDS-PAGE and analyzed by immunoblot for Ac3K9/14 H3. [file 1471-2121-11-81-S8.TIFF]

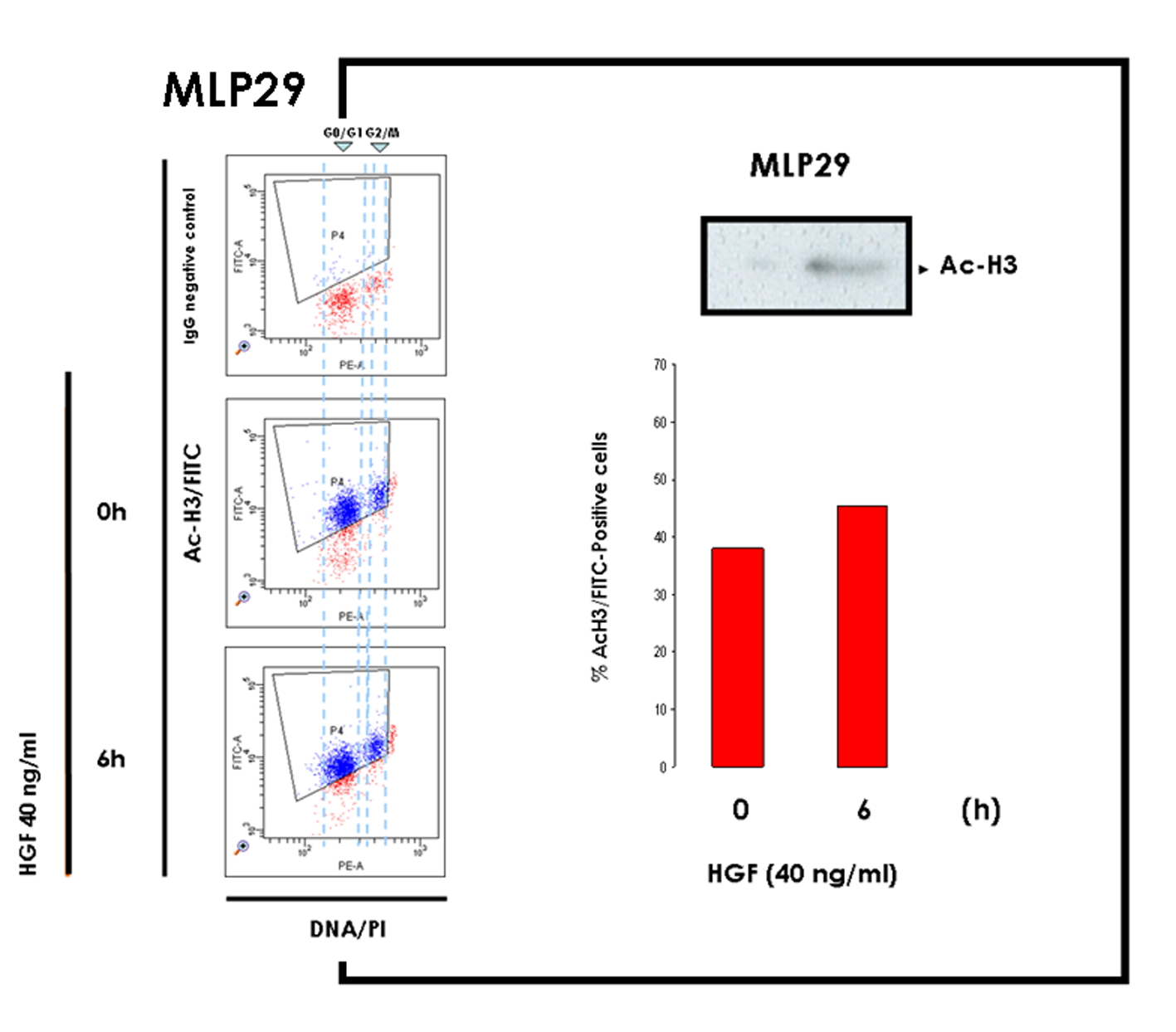

Supplement: Additional file 9 — Flow cytometric and immunoblot analyses of Ac3K9/14 H3 in the context of DNA content of MLP29 cells treated with HGF/SF1. Bivariate cytograms indicate the levels of expression of Ac3K9/14 H3 in each sub-compartment of the cell cycle. Bar graphs show the percentage of FITC-positive cells. The data presented are representative of three independent experiments. For immunoblot analysis, treated MLP29 cells were lysed and total protein (50 μg) was resolved by SDS-PAGE and analyzed by immunoblot for Ac3K9/14 H3. [file 1471-2121-11-81-S9.TIFF]
